# Accumulation of Elements in Biodeposits on the Stone Surface in Urban Environment. Case Studies from Saint Petersburg, Russia

**DOI:** 10.3390/microorganisms9010036

**Published:** 2020-12-24

**Authors:** Katerina V. Sazanova (nee Barinova), Marina S. Zelenskaya, Vera V. Manurtdinova, Alina R. Izatulina, Aleksei V. Rusakov, Dmitry Yu. Vlasov, Olga V. Frank-Kamenetskaya

**Affiliations:** 1 Saint Petersburg State University, University Emb., 7/9, 199034 Saint Petersburg, Russia; marsz@yandex.ru (M.S.Z.); alina.izatulina@mail.ru (A.R.I.); alex.v.rusakov@gmail.com (A.V.R.); dmitry.vlasov@mail.ru (D.Y.V.); o.frank-kamenetskaia@spbu.ru (O.V.F.-K.); 2 Komarov Botanical Research Institute of Russian Academy of Science, Professor Popov Street, 2, 197376 Saint Petersburg, Russia; 3 The Archive of the Russian Academy of Sciences, University Emb., 1, 199034 Saint Petersburg, Russia; 4 State Museum of Urban Sculpture, Nevsky Prospect 179, 191167 Saint Petersburg, Russia; ver4ik21@mail.ru

**Keywords:** biodeposits, microbial biomineralization, mosses, fungi, algae, lichens, rocks, environment

## Abstract

The pattern of elements accumulation in biodeposits formed by living organisms and extracellular products of their metabolism (biofouling, primary soils) on different bedrocks (of the monuments of Historical necropoleis in Saint Petersburg) were studied by a complex of biological and mineralogical methods (optical microscopy, SEM, EDX, XRD, ICP MS, XRFS). The content of 46 elements in biodeposits with various communities of microorganisms is determined. The model recreating the picture of the input and selective accumulation of elements in biodeposits on the stone surface in outdoor conditions is assumed. It is shown that the main contribution to the elemental composition of biodeposits is made by the environment and the composition of the microbial community. The contribution of leaching under the action of microbial metabolites of mineral grains, entering biodeposits from the environment, is significantly greater than that of the underlying rock.

## 1. Introduction

Natural stone located in the urban environment undergoes intensive biological colonization [1,2,3,4]. The lithobiontic microbial community (fungi, algae, bacteria, mosses, and lichens) inhabiting the stone surface interacts with the underlying rock, which leads to additional weathering of stone and organomineral biodeposits (biofouling, young soils) formation on the stone surface, containing organic substances, products of bedrock weathering, as well as various elements getting from the environment (air, soil etc) in addition to the organisms themselves [5]. We use the term biodeposits, meaning the complex and different nature of the accumulation of biological objects and products of their vital activity on a solid substrate [6]. Biodeposits include biofouling (microorganisms, lichens, mosses), as well as primary soils under mosses together with organic and mineral components from the outdoor environment and the underlying substrate.

Under the influence of aggressive metabolic products, extracellularly secreted by microorganisms, primarily organic acids, bedrocks dissolve, which contributes to the leaching of microelements from rock, their accumulation in biodeposits, and an increase in their mobility and bioavailability. Probably, biodeposits can also accumulate elements, including heavy metals, leached by the products of microbial metabolism from the grains of minerals that have entered them from the environment. If there is oxalic acid (secreted by many fungi, lichens, as well as a number of bacteria) among the metabolic products, elements accumulating in biofilms can react with it, which leads to the formation of oxalic acid salts, primarily calcium oxalates [3,7,8,9,10,11,12,13,14,15,16,17,18]. Unlike the organic component of biodeposits heavy metals are not biodegradable and can transfer through the food chain and are susceptible to bioaccumulation [19]. 

It is known that the accumulation of elements by the microbial community can be carried out extracellularly and intracellularly [8,9,10,19,20,21]. Numerous data indicate an important role of the sorption mechanism on the surface of cell walls when the accumulation of cations by bacteria, microalgae, microfungi, lichens, and mosses [22,23,24,25,26,27,28,29,30]. The cell wall can take part in the sorption of ions even in the absence of physiological activity (as dead biomass) [19]. The extracellular accumulation of cations can be accompanied by ion exchange [20], leading to the formation of complexes in which the cations are coordinated by the organic functional groups of the cell walls [21]. In addition, extracellular accumulation of elements in biodeposits formed by lichens and fungi can occur through the binding of cations with small organic molecules, primarily organic acids. Organic acid production activity (oxalic, citric, malic, gluconic, fumaric, succinic and some others) is a characteristic of many species of fungi and some lichens [31,32,33]. 

Intracellular accumulation of elements can occur through the absorption of metals as a result of the work of transport systems. Once in the cell, the metal can be immobilized inside the vacuoles in the cytoplasm. The mechanisms of metal immobilization can involve organic acids or specific proteins (metallothioneins and phytochelatins capable to bind metals through SH-groups) [9,10,19,34].

According to [35], higher concentrations Al, Cr, Fe, Mn, Ni, Ti are recorded in mosses, and Hg, Cd, Pb, Cu, V, Zn in lichens. Apparently, the concentrations of most elements in mosses are more dependent on the chemical composition of rainwater fallen over a short period, rather than over the whole season, as in the case of lichens [36].

The known features of the accumulation of metals and other elements by organisms of various taxonomic and ecological groups are actively used in biotechnology. Mosses and lichens are used for bioindication [37,38,39,40]. Fungi, due to their ability to hyperaccumulate metals in concentrations exceeding those in the environment, are more often used for bioremediation than other organisms [19]. In addition, fungi have a high degree of resistance to the action of heavy metals, which allows them to survive in an environment with a high concentration of metals and utilize the physiologically active mechanisms for their accumulation [22,23,24].

Thus, the mechanisms of metal accumulation by organisms of various taxonomic and ecological groups are described in detail in the literature and are being actively studied. However, in natural habitats, biodeposits are usually formed by communities of organisms. There is practically no data on the bioaccumulation of elements in biodeposits at the community level, which does not allow for a complete understanding of the patterns of accumulation of inorganic elements on the surface of different rocks and of the geochemistry of the biodeposits present on them.

In this work we are trying to move forward in this direction and identify a pattern of accumulation of elements in biodeposits on the stone surface in urban environment (case of Saint Petersburg). In particular, we planned to: (a) determine the elemental composition of biodeposits with various species composition of microorganisms on different bedrocks; (b) carry out a comparative analysis of the influence of the bedrock, the environment and the species composition of microorganisms on biodeposit elemental composition; (c) restore (at a model level) the picture of the input and selective accumulation of elements in biodeposits in outdoor environment.

## 2. Materials and Methods

### 2.1. Sampling

**Figure 1:**
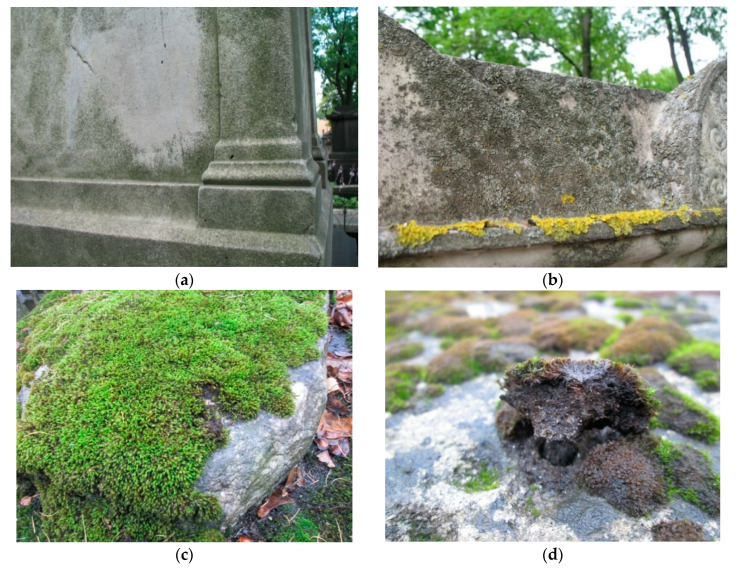
The main types of biodeposits on the surface of stone monuments of the historical necropoleis: (**a**)—biofilm with a predominance of microscopic fungi and algae; (**b**)—biofilm with a predominance of lichens; (**c**)—vegetative biomass of a moss; (**d**)—primary soil under the moss cover.

Biodeposits were collected from the surface of stone monuments of the Historical necropoleis located in the central part of Saint Petersburg. Here, in a small area, there are monuments made of various types of decorative stone, which are practically in the same outdoor environment and undergo biological colonization. The following types of biodeposits containing various communities of living organisms from the surface of different rocks were collected (Table 1, Figure 1): I—biofilms with a predominance of microscopic fungi and algae; II—biofilms with a predominance of lichens; III—vegetative biomass of the moss; IV—primary soil under the moss cover. Although the composition of lichens on the surface of stone monuments includes both crustose and foliose forms we took only samples of foliose lichens, since they have a large biomass, are relatively easy to take from the surface, and also create special conditions for the development of accompanying microorganisms, such as, for example, fungi. 

The structural and textural features as well as the mineral composition of the rocks of the monuments of the necropoleis were previously investigated by us, and on this basis, assumptions about their origin were made [41]. Using this data we selected underlying rocks (granites, marbles, limestones) differing in mineral and elemental composition, as well as in their petrographic characteristics (primarily homogeneity and porosity) (Table 1 and Table 2), i.e., properties significantly influencing the intensity of weathering and the rate of diffusion of elements from the bedrock into the biodeposits on it surface. When selecting biodeposits we used their morphological properties, studied by us earlier [42].

In order to reliably reveal the influence of the underlying stone substrate on the elemental composition of biodeposits, we also took two biodeposist samples from an eroded surface of a wooden monument for contrast (one with a predominance of lichens and the other with mosses). In addition, in order to compare the elemental composition of dust with the elemental composition of the rock on which it was formed and the compositions of biodeposits on the same rock, we took a dust sample from the surface of a homogeneous white marble.

The collected material was studied using a complex of biological and mineralogical methods. 

### 2.2. Study of Biodiversity 

All samples of biodeposits were characterized by their appearance and dominant species of organisms. The identification of microscopic fungi was carried out by isolating them into a pure culture. For primary isolation, maintenance in culture and identification of micromycetes, Czapek-Dox (HiMedia) culture medium was used. Small fragments of biodeposits were placed on the surface of the nutrient medium in Petri dishes. In addition, washings from the substrate surface were used for inoculation. The resulting cultures were incubated in a thermostat for 2–4 weeks at a temperature of 25 °C until sporulation appearance, after which microscopy and identification by morphological characteristics were carried out in accordance with guidebooks and monographs [43,44,45,46]. The species were verified in accordance with the modern nomenclature using the Index Fungorum electronic database [47]. 

The identification of algae was carried out by morphological characteristics. For this purpose, we performed direct microscopy of samples (by means of Leica DM1000 microscope) after settling in distilled water for a week. To determine the species composition, identifiers and monographs [48] as well as the electronic database AlgaeBase [49] were used.

Identification of lichens was carried out according to the generally accepted method using identifiers [50,51,52], by means of the Leitz Laborlux S microscope, and stereomicroscope MBS-10. The nomenclature of lichen species has been brought in line with the list of lichens in Scandinavia [50].

Identification of bryophytes was carried out according to morphological characteristics using identifiers [53,54], by means of the Micromed-2 microscope and stereomicroscope MBS-10. The nomenclature of bryophytes has been brought in line with the list of bryophytes in Europe, Macaronesia and Cyprus [55]. 

### 2.3. Study of the Mineral Component of Biodeposits

The study of the phase composition of mineral grains in biodeposits was carried out via X-ray powder diffraction by means of Bruker “D2 Phaser” powder X-Ray diffractometer operated with CoKα radiation. X-ray diffraction patterns were collected at room temperature in the range of 2θ =5–70° with a step of 0.02° 2θ and a counting time of half second per data point. A sample holder from a single crystal silica slice was used to eliminate the background noise. Phase identification was carried out using the ICDD PDF-2 database (release 2016). 

To determine the phase composition of secondary silicate minerals with a high degree of dispersion, which are part of primary soils (type IV of biodeposits), we used oriented preparations obtained by successive precipitation of heavy and light mineral fractions [56].

The distribution and qualitative elemental composition of mineral grains in biodeposits were determined via scanning electron microscopy (SEM) and energy-dispersive X-ray spectroscopy (EDX) methods, respectively. Measurements were performed by means of the Desktop Scanning Electron Microscope TM3000 (Hitachi), which was equipped with OXFORD energy dispersive microanalysis attachment and secondary electron (Everhart-Thornley, UK) detector based on the highly sensitive YAG crystal with the resolution of 0.1Z of the atomic number. The specimens were coated with carbon (~15 nm). Magnification range varied from 100x to 1000x. The EDX spectra were analyzed by means of the EDAX Genesis software package (semiquantitative analysis was performed by standard-less method that is generally reliable for elements with Z > 10).

### 2.4. Quantitative Study of the Elemental Composition of Biodeposits

The main method used for the quantitative determination of the elemental composition of collected bioformations was inductively coupled plasma mass spectrometry (ICP MS). This method was used to determine the content of 37 elements (Na, Mg, Al, K, Ti, Mn, Be, V, Cr, Co, Ni, Cu, Zn, Ga, Rb, Sr, Y, Zr, Nb, Mo, Ag, Sn, Sb, Cs, Ba, La, Ce, Pr, Nd, Sm, Eu, Gd, Tb, Dy, Ho, Er, Tm, Yb, Lu, Hf, W, Th, U) in all biodeposits. The solutions were prepared via two different methods of decomposition: complete acid breakdown and fusion with lithium metaborate [57]. The solution analysis was carried by means of ELAN-DRC-e and Agilent 7700x spectrometers using a computer data processing program which automatically accounts for both isotopic and molecular overlays on mass-spectral analytical lines of the determined elements (analyst G.A. Oleinikova).

The content of Ca, Si and Fe in biodeposits of III and IV types (in mosses and primary soils) was determined by X-ray fluorescence method by means of a vacuum X-ray fluorescence crystal-diffraction scanning spectrometer “SPECTROSCAN MAKS-GV” (Russia). The samples were preliminarily dried in a drying oven at a temperature of 70 °C, crushed, ground to the size of a powder, weighed 2g each, and was pressed into tablets using a hydraulic press.

The determination of Ca, Si, and Fe in biodeposits of types I and II (with a predominance of microscopic fungi + algae and lichens, respectively) due to insufficient amount of material was performed via electron probe microanalysis by means of the scanning electron microscope Camscan-4 equipped with X-ray energy microanalyzer AN-10000 (UK) at a 30 kV acceleration voltage and 2 lm electron beam diameter (analyst Yu. L. Kretser). SEM Calibration Specimens (registered standard number 1413) from Microanalysis consultants Ltd. were used as standards. In order to obtain data on the average composition of the sample, the analysis was carried out in the scanning mode at a magnification of x200. Data processing was performed by means of the ZAF-4/FLS program. 

### 2.5. Statistical Analysis

Statistical analysis was performed via the student’s t-test and principal component analysis (PCA) using Microsoft Excel, MetaboAnalyst and Origin Pro software. 

## 3. Results

### 3.1. Microorganism Species Composition of Studied Biodeposits

Microscopic fungi were present in all types of biodeposits, but in the first type, micromycetes, together with algae, formed the basis of the community, while in other types of biodeposits they were represented by individual colonies as mycological analysis showed. The species composition of fungi included: *Alternaria alternata* (Fr.) Keissl., *Alternaria chartarum* Preuss, *Arthrinium phaeospermum* (Corda) M.B. Ellis, *Aureobasidium pullulans* (de Bary and Löwenthal) G. Arnaud, *Botrytis cinerea* Pers., *Cladosporium cladosporioides* (Fresen.) G.A. de Vries, *Cladosporium herbarum* (Pers.) Link, *Cladosporium sphaerospermum* Penz., *Coniosporium* sp., *Didymella glomerata* (Corda) Qian Chen and L. Cai, *Epicoccum nigrum* Link, *Exophiala exophialae* (de Hoog) de Hoog, *Fusarium oxysporum* Schltdl., *Mucor hiemalis* Wehmer, *Paecilomyces divaricatus* (Thom) Samson, Houbraken and Frisvad, *Penicillium brevicompactum* Dierckx, *Penicillium herquei* Bainier and Sartory, *Phaeosclera* sp., *Phialophora asteris* (Dowson) Burge and I. Isaac, *Phoma herbarum* Westend., *Scytalidium lignícola* Pesante, *Talaromyces purpureogenus* Samson, N. Yilmaz, Houbraken, Spierenb., Seifert, Peterson, Varga and Frisvad, *Trichoderma viride* Pers., *Trichocladium griseum* (Traaen) X. Wei Wang and Houbraken.

Most often, dark-colored microscopic fungi *A. alternata*, *Aureobasidium pullulans*, *C. cladosporioides*, which dominated in type I together with algae, was constantly observed in all other types of biodeposits. Algae in type I were represented by species of *Chlorophyta*, mainly genera *Trentepohlia*, *Trebuxia*, and *Desmococcus*. In addition, diatoms were quite commonly found on SEM images.

Lichens dominating in type II biodeposits were represented by the species: *Physcia tenella* (Scop.) DC., *Physconia distorta* (With.) J.R.Laundon, *Candelariella aurella* (Hoffm.) Zahlbr., *Myriolecis crenulata* (Hook.) Śliwa et al., *Myriolecis invadens* (H. Magn.) Śliwa et al., *Xanthoria parietina* (L.) Th. Fr., *Phaeophyscia orbicularis* (Neck.) Moberg, *Verrucaria muralis* Ach.

The species composition of mosses in type III biodeposits included *Brachythecium salebrosum* (Hoffm. ex F. Weber and D. Mohr) Schimp., *Ptychostomum pseudotriquetrum* (Hedw.) J.R. Spence and H.P. Ramsay ex Holyoak and N. Pedersen, *Bryoerythrophyllum recurvirostrum* (Hedw.) P.C. Chen, *Ceratodon purpureus* (Hedw.) Brid., *Schistidium apocarpum* (Hedw.) Bruch and Schimp., *Marchantia polymorpha* L. Common for carbonate and silicate rocks were the species: *S. apocarpum*, *B. salebrosum*, *P. pseudotriquetrum*. *B. recurvirostrum* was found only on Pudozh stone and granite, while *C. purpureus* was found only on marble and wood.

### 3.2. Phase Composition of Mineral Component of Studied Biodeposits

**Figure 2:**
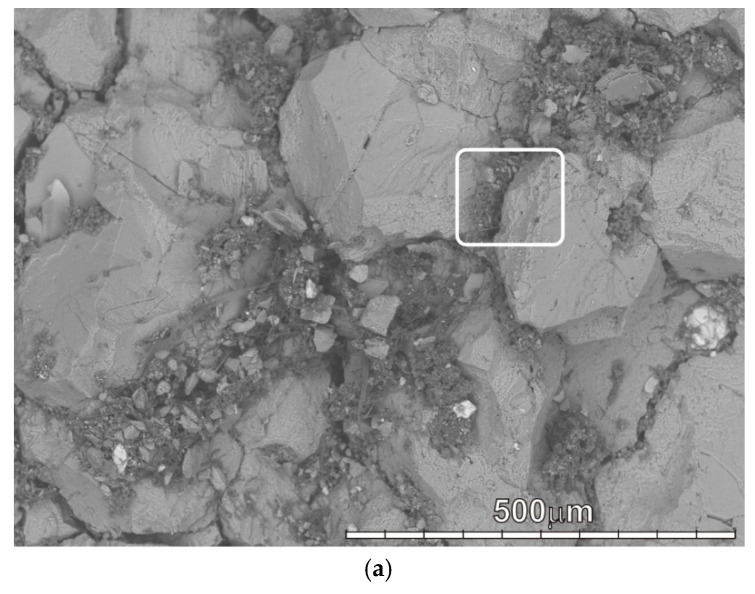

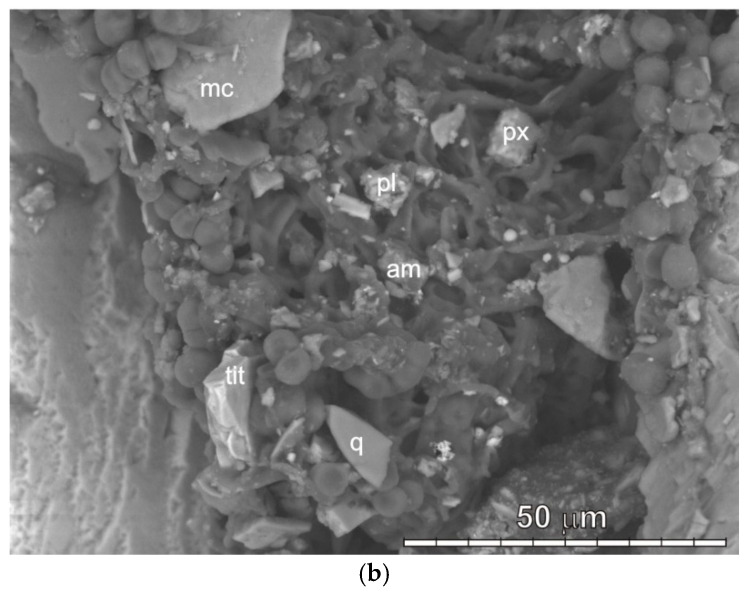
SEM-image of a biofilm with a predominance of microscopic fungi on the surface of a homogeneous calcite marble: (**a**)—microcolonies of fungi around calcite grains (the white frame shows the area shown in figure b); (**b**)—grains of various silicate minerals among fungal hyphae and rounded cells (in the region shown in Figure 2a): quartz (q), plagioclase (pl), mica (mc), pyroxene (px), Fe—titanite (tit), Pb—amphibole (am).

**Figure 3:**
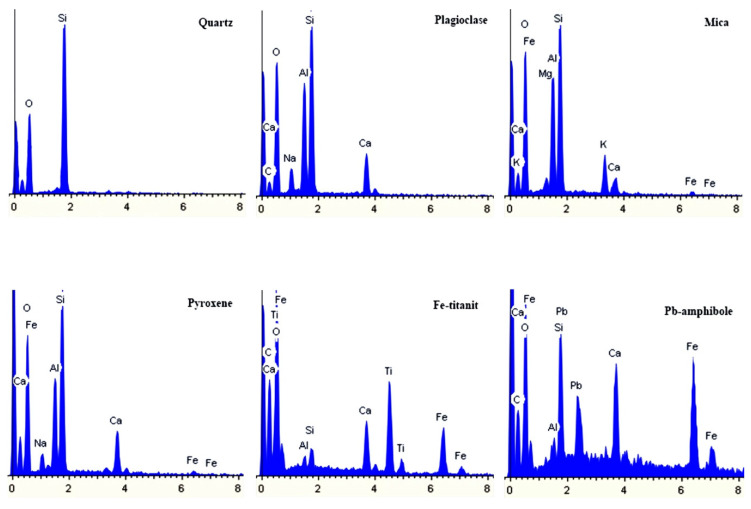
Examples of EDX-spectra of silicate minerals grains in a biofilm with a predominance of microscopic fungi on the surface of a homogeneous calcite marble.

**Figure 4:**
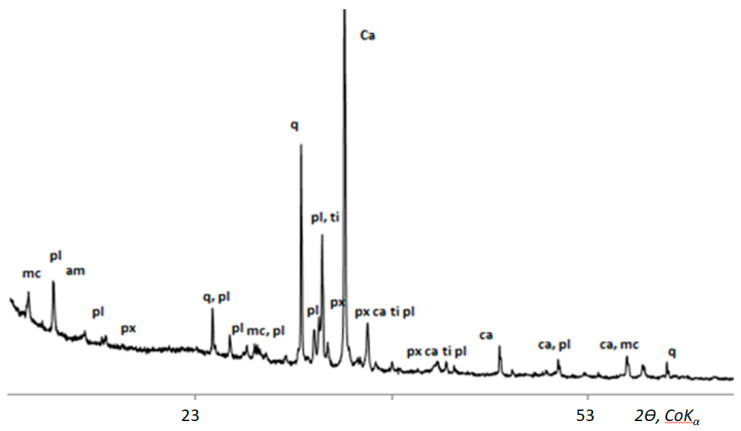
X-ray diagram of silicate minerals grains in biofilm with a predominance of microscopic fungi on the surface of a homogeneous calcite marble: quartz (q), calcite (Ca), plagioclase (pl), mica (mc), pyroxene (px), titanite (ti), amphibole (am).

The results of the study showed that in biodeposits of types I, II, and III, we can find almost all minerals of rocks from which the monuments of the necropolies are made [41]. The most common minerals are typical for granites and other silicate rocks: quartz, feldspars (acidic plagioclase, microcline), pyroxenes, amphiboles, biotite, etc, rocks from which most of the stone monuments of the necropolies were made. The mineral composition of grains varies very little from site to site and does not depend on the underlying rock. This is clearly seen in the example of a biofilm with a predominance of microscopic fungi on the surface of a homogeneous fine-grained calcite marble, in which grains of numerous silicate minerals are present (Figure 2, Figure 3 and Figure 4).

In all the samples of primary soils, in addition to minerals found in biofouling there also are secondary layered silicates present in different proportions: mica (biotite, polytype 1M), magnesian chlorite, and disordered mixed-layer silicate of the mica-montmorellonite type.

### 3.3. Elemental Composition of Biodeposits (Quantitative Data)

**Figure 5:**
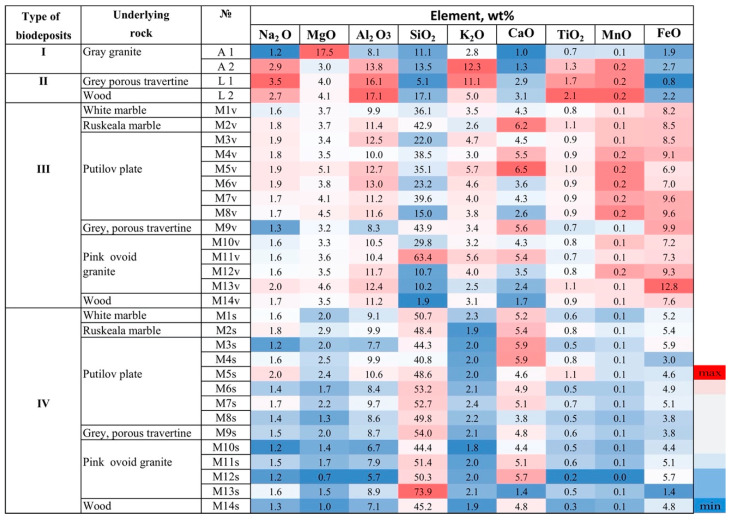
Heat map of the main impurity elements (wt%) in the biodeposits on the surface of the different rocks, (ICP MS analysis data).

**Figure 6:**
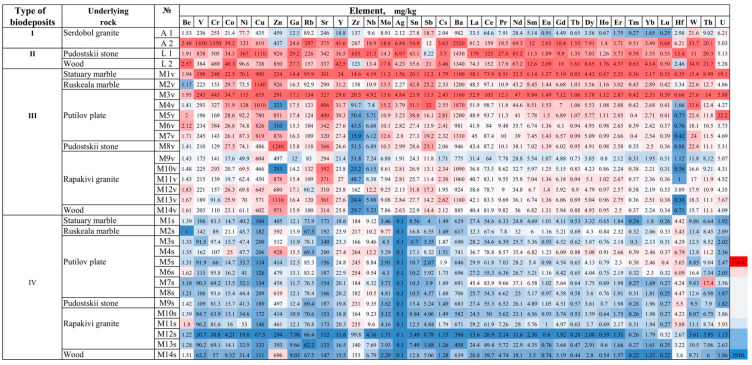
Heat map of trace impurity elements (mg/kg) in the biodeposits on the surface of the different rocks (1 mg/kg = 10^−5^ wt%).

46 elements were determined in the studied biodeposits, they can be divided into 2 groups—the main impurity elements, the content of which is not less than 0.2 wt% (in oxide form not less than 0.1 wt%, Figure 5, Table 3) and trace elements, the content of which is less than 0.2 wt% (Figure 6, Table 4). 

The various types of biodeposits are characterized by an identical set of inorganic elements but differ in their quantity.

The content of the main impurity elements Na, Al, Ti, and Mn is higher in biodeposits with a predominance of lichens (type II) in comparison with other types of biodeposits (Figure 5, Table 3). The content of silicon and calcium is significantly higher in mosses and, especially, in the primary soil under the moss cover. Iron is contained in the vegetative part of mosses and the underlying soil (in type III and IV of biodeposits) in a higher concentration than in type I and II of biodeposits. But in contrast to calcium and silicon, the iron content is higher in the vegetative part of mosses (type III of biodeposits), and not in the primary soil (type IV of biodeposits).

Trace elements are found in higher concentrations in biodeposits dominated by fungi and algae, as well as lichens (type I and II of biodeposits) than in the primary soil with a moss cover (type III and IV of biodeposits) (Figure 6, Table 4). Lichen-dominated biodeposits (type II) are characterized by the accumulation of most trace impurity elements in the highest concentrations. The concentration of most trace impurity elements was higher in the vegetative part of mosses (type III of biodeposits) as compared to the primary soil under them (type IV of biodeposits). The exceptions were Zr, Nb, Hf, the concentration of which was higher in the primary soil.

No relationship was found between the elemental compositions of the underlying rock and biodeposits (Figure 5 and Figure 6). No significant differences were observed when comparing biodeposits on silicate and carbonate rocks in terms of the content of calcium and silicon, as well as when comparing the primary soil on monuments made of wood and stone. Only the content of Si in the vegetative part of moss (III type) sampled from the surface of stone monuments was higher than its content in moss sample taken from the surface of the wooden monument. In primary soil (IV type) Si content did not differ significantly between all samples. Similar data was obtained for calcium: its content in mosses on a wooden monument was no more than 4-6 times lower than on the surface of a stone, and in the primary soil was almost equal.

As shown by the example of white calcite homogeneous marble (Table 5), the content of the main impurity elements in biodeposits and dust is significantly higher (not less than an order of magnitude) than in the bedrock. The concentrations of these elements in biodeposits and dust are of the same order of magnitude. At the same time, a trend can be seen: the concentration of impurity elements in the dust is closer to the concentrations in the primary soil (in comparison with the vegetative part of mosses).

**Figure 7:**
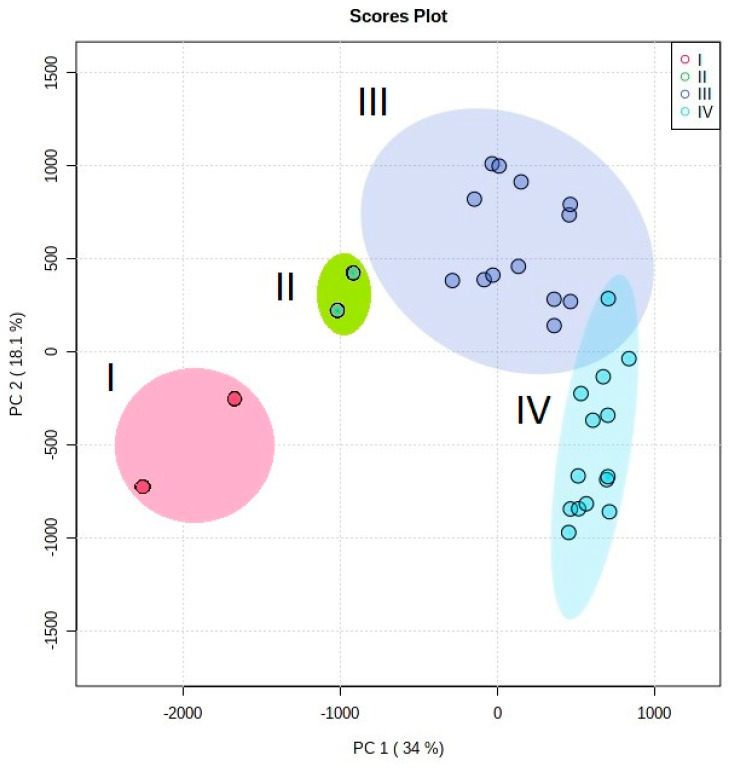
Results of principal component analysis (PCA) of the main impurity elements in biodeposits on the surface of stone: I—bifilms with a predominance microscopic fungi and algae; II—biofilms with a predominance lichens; III—vegetative biomass of the moss; IV—primary soil under the moss cover. The main contribution to the statistical model for PC 1 is made by TiO_2_, Na_2_O, Al_2_O_3_ and for PC 2 by MgO, K_2_O, and MnO.

**Figure 8:**
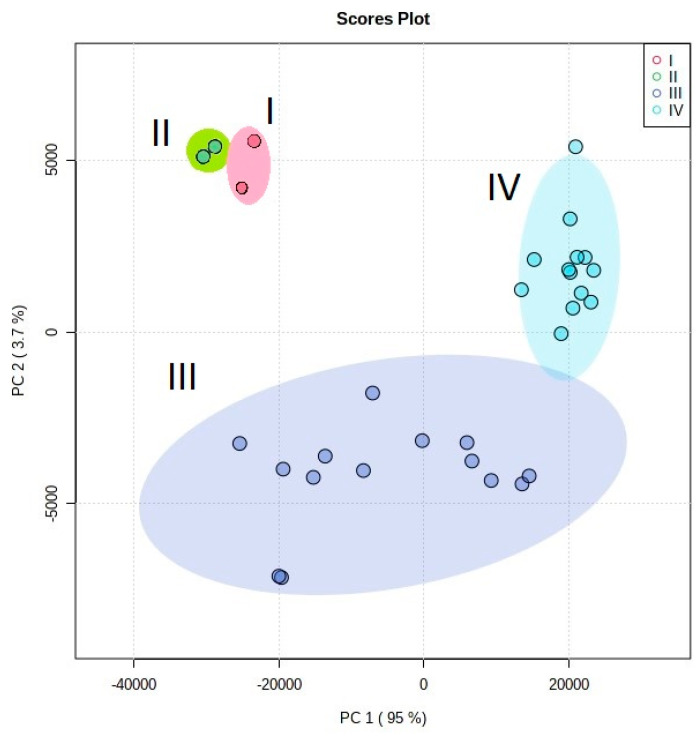
Results of PC analysis of the trace elements in biodeposits on the surface of stone: I—biofilms with a predominance microscopic fungi and algae; II—biofilms with a predominance lichens; III—vegetative biomass of the moss; IV—primary soil under the moss cover. The main contribution to the statistical model for PC 1 is made by Eu, Sm, Pr, Nd, Ce, Gd, Zn, Rb, Dy, Sn and for PC 2 by Lu, Sr, Y, Pb, Ge, Ag, Cs, Ba, Zr, Ni.

Analysis of the studied elemental compositions by the method of principal components (PCA) leads to the formation of four clusters corresponding to all types of analyzed biodeposits and makes it possible to assess the degree of difference in their compositions (Figure 7 and Figure 8). 

**Figure 9:**
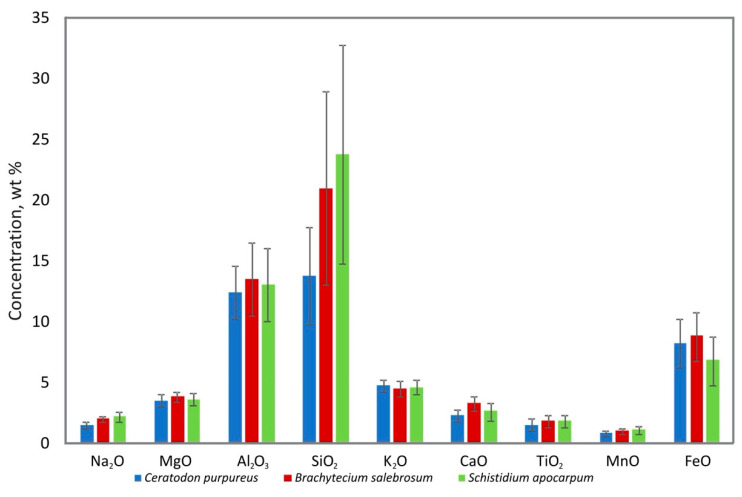
Content of the main impurity elements (wt%) in mosses of different species composition.

Unlike the I and II types, macrodeposists of moss (III and the IV types) can be analyzed at the species level, which can also be important when assessing the specificity of the accumulation of elements by organisms. Moss samples (type III of biodeposits), represented by only one species, totaled a representative set (more than three samples) for the species *C. purpureus*, *B. salebrosum*, and *S. apocarpum*. Analysis of the distribution of the main impurity elements depending on the belonging of the mosses to specific species showed significant differences in the accumulation of silicon (Figure 9). The amount of Si in the biomass increased in the series *C. purpureus*, *B. salebrosum, S. apocarpum*. 

## 4. Discussion

The obtained results allow to make a comparative analysis of the influence of the bedrock, the environment, and the species composition of microorganisms on the elemental composition of biodeposits and restore (at a model level) the picture of the input and selective accumulation of elements in biodeposits on the surface of the different rocks in an outdoor environment. 

### 4.1. The Influence of the Bedrock, the Environment, and the Species of Microorganisms on the Elemental Composition of Biodeposits

**Figure 10:**
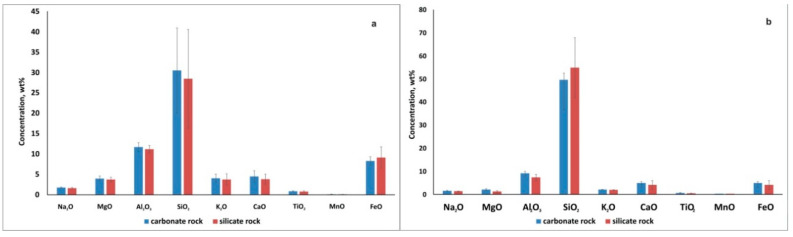
Comparison of average concentrations (wt%) of the main impurity elements in biodeposits on carbonate and silicate rocks: (**a**)—in mosses (III type of biodeposits); (**b**)—primary soil under them (IV type of biodeposits)**.**

No significant differences in the elemental composition of biodeposits depending on the underlying substrate were revealed (Figure 5 and Figure 6). For example, this is clearly seen when comparing biodeposits on silicate and carbonate rocks in terms of the content of calcium and silicon (Figure 10).

This is also confirmed by the closeness of the elemental compositions of biodeposits on the surface of wood and rocks (Figure 5 and Figure 6).

Accordingly, it can be assumed that the contribution of the underlying rock to the elemental composition of the studied biodeposits is significantly less than that of the environment. This conclusion is also supported by the fact that the content of impurity elements in the rocks is at least an order of magnitude lower than in the deposits on its surface (Table 5). There is also evidence of that in the results of a comparison of the elemental composition of type III and IV biodeposits (mosses and primary soil formed under them). The results of cluster analysis (Figure 7 and Figure 8) indicate the decisive role of the taxonomic affiliation of organisms in the pattern of the selective accumulation in biodeposits of elements transferred to the stone surface predominantly from the environment. The elemental composition of type I biodeposits (with a predominance of fungi and algae) differs significantly from type II biodeposits (with a predominance of lichens) only in terms of the main impurity elements (Figure 7). Differences in the elemental composition of biodeposits of types III and IV (the upper vegetation part of the mosses and the lower layer of the primary soil) are more pronounced for the trace elements (Figure 8).

Thus, the results of the comparative analysis showed that the elemental composition of investigated biodeposits on the surface of outdoor stone is mainly controlled by the environment and the composition of microorganism species inhabiting stone surface (at the level of large taxa). The specificity in the accumulation of elements at the species level (as shown by the example of mosses) also takes place, but it is not as contrasting as when comparing large taxa.

### 4.2. The Picture of the Input and Accumulation of Elements in Biodeposits on the Surfaces of Stone Monument in Outdoor Environment (at a Model Level)

The obtained results and knowledge gathered previously by us and other researchers allow us to recreate at a model level the picture of the input and selective accumulation of elements in biodeposits on the stone surfaces. The lack of relationships between the elemental composition of biodeposits and underlying rocks can be explained by assuming that a significant contribution to the elemental composition of biodeposits is made by leaching under the action of microbial metabolites of mineral grains present in biofoulings and young soils. According to our data, the mineral composition of the grains in the biodeposits is determined by the mineral composition of all the rocks from which the monuments of the necropoleis are made and varies little from monument to monument. The results of our long-term monitoring indicate intensive weathering of the stone materials of the necropoleis monuments [41]. Grains of various minerals entering the environment can be picked up by wind currents, mixed and have a tendency to be averaged over all the stone monuments of the necropoleis.

Of course, elements turn out to be on the surface of monuments and without the participation of wind, for example, in the form of aerosols directly from the air during acid rains and fogs [41]. However, the mineral composition of grains in biodeposits on the surface of monuments, which was determined by us, can be formed only with the participation of wind flows. Thus, all the results obtained indicate that elements enter the biodeposits on the stone surface (biofoulings and primary soils) mainly from the environment. This conclusion correlates with a number of other researches [40,58].

The differences in the elemental composition of the studied biodeposits via cluster analysis (Figure 7 and Figure 8) can be explained using the well-known mechanisms of accumulation of elements by the microbial community. Biodeposits of types I and II (in which the fungal component dominates) are characterized by a greater ability to accumulate elements (with the exception of iron, calcium, and silicon prevailing in mosses). This can be explained by the fact that fungal biomass is more capable of sorption of cations due to the large number of highly active functional amino groups in chitin [59], the presence of extracellular melanins [31], as well as due to the significant contribution of the cell wall to the total biomass of fungi [19]. Because sorption is a surface reaction, the biosorption potential of a biosorbent depends on its surface area and its polarity. We can say that the performance of a biosorbent depends on the ionic state of the biomass. Thus, fungal biomass received much attention as a biosorbent because of the presence of a high percentage of cell-wall material, which increases the variety of functional groups involved in metal binding [60]. Additionally, it is impossible to exclude the formation of complexes occurring due to extracellular ion exchange reactions and intracellular accumulation of elements due to their binding by specific proteins and organic acids.

**Figure 11:**
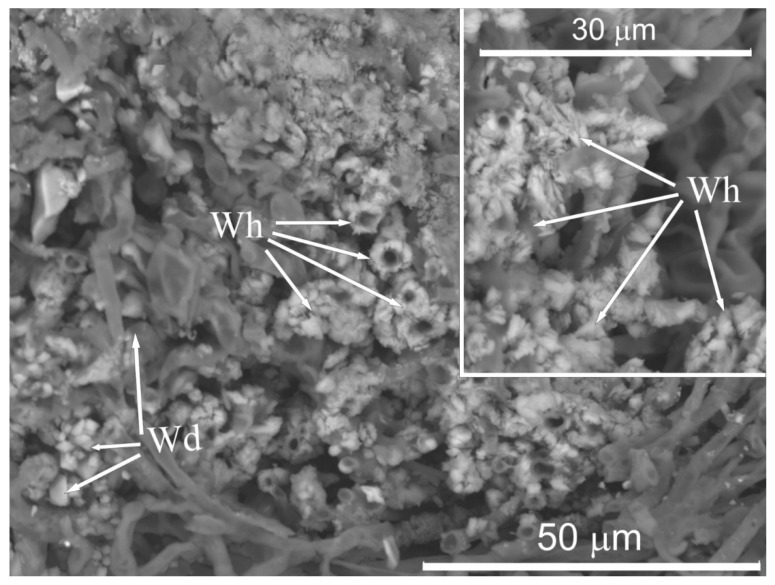
SEM-images of calcium oxalate crystals in lichen dominated biodeposits on the surface of homogeneous calcite marble: dipyramidal crystals of weddellite (Wd) and lamellar crystals of whewellite (Wh).

Another possible reason for determining the composition of ions in biodeposits is the extracellular formation of poorly soluble salts of oxalic acid, primarily calcium oxalates (Figure 11). Oxalic acid is excreted by many species of lichen [18]. Besides, among the fungi revealed in the studied biodeposits, the species of genus *Penicillium* have an intense ability for extracellular production of organic acids, including oxalic acid. Some other species, for example, *T. viride, A. alternata, A. pullulans*, are also capable of acid production activity under certain conditions [61,62].

Differences in the content of the main impurity elements of biodeposits of types I and II were clear (Figure 7), and they were really absent for trace impurity elements (Figure 8). Perhaps this is due to the fact that for the main impurity elements the role of the mechanism of intracellular accumulation is more significant than for the trace impurity elements, and organisms can exhibit greater selectivity and variability in the accumulation of these elements.

Mosses, regardless of their species, are less likely to accumulate most elements, with the exception of iron, calcium, and silicon. We believed that in mosses, the extracellular fraction of metals is usually easily exchanged and tends to reflect current environmental conditions and sporadic pollution by many elements. The intracellular fraction is the result of the integration of metals over a longer period of time and thus characterizes the average situation in the environment [63]. The revealed regularity can be explained if we assume that the elements present in the highest concentrations (iron, calcium and silicon) accumulate in mosses intracellularly, and the elements with lower concentrations mainly bind extracellularly.

The primary soil under mosses (type IV biodeposits) contains the lowest concentrations of elements, with the exception of Ca, Si, Hf, Nb, and Zr. Kłos et al. [64] proposed two mechanisms for the transfer of metals from the soil to mosses: transport of elements with raised dust and their diffusion through aqueous solutions. It can be assumed that the elements transferred by aqueous solutions into the plant biomass of mosses come mainly from primary soil, to where they get from mineral grains from the environment and, to a lesser extent, from the underlying rocks. Ca and Si, probably, cannot be consumed by mosses in such high concentrations and therefore dominate in primary soil. As for the trace impurity elements Hf, Nb, and Zr, the probable reason for their predominant accumulation in the primary soil, rather than in the vegetative part of the moss, is probably due to the poor bioavailability of these elements for the plant. Some evidence suggests that only a small fraction of zirconium is available for plant uptake, because of strong binding with organic and inorganic ligands in soils [65].

## 5. Conclusions

The studied biodeposits formed by living organisms and the extracellular products of their metabolism on the stone surface in outdoor conditions (biofouling, primary soils) contain numerous elements (from Be to REE), the concentration of which varies substantially.

The element composition of biodeposits is controlled mainly by the environment and the species composition of microorganisms inhabiting stone (at the level of large taxa). The contribution to the elemental composition from leaching (under the action of microbial metabolites) of mineral grains, which enter biodeposits from the environment, is more significant than that of the underlying rocks.

The picture of the input and selective accumulation of elements in biodeposits on the stone surfaces (recreated at a model level) allows us to explain the insignificant contribution of the underlying rocks to the elemental composition of biodeposits only if we take into account the essential role of wind flows in the formation of the biodeposit mineral component.

The obtained results significantly expand the understanding of the chemical composition of the medium in which oxalate crystallization occurs in biofilms and contribute to the development of ideas about microbial biomineralization mechanisms.

## Figures and Tables

**Table 1:** List of the biodeposits samples collected from Saint Petersburg necropoleis monuments.

Sample	Monument	Underlying rock
I type. Biodeposits with a predominance of microscopic fungi and algae		
A 1	A. E. Martynov, NMA ^1^	Grey homogeneous granite
A 2	M. P. Zotova, N-18 ^2^	
II type. Biodeposits with a predominance of lichens		
L 1	E. H. Minich, N-18	Grey porous travertine
L 2	B. M. Kustodiev, NMA	Wood
III and IV types. Vegetative biomass of the moss and primary soil under the moss cover.		
M1v ^3^, M1s ^4^	M. S. Zotova, N-18	White homogeneous marble
M2v, M2s	E. A. Rummel, N-18	Grey-white banded heterogeneous marble
M3v, M3s	T. D. Von-Fewson, N-18	Stratified limestone
M4v, M4s	G. I. Ogarev, N-18	
M5v, M5s	I. A. Myasnikov, N-18	
M6v, M6s	T. A. Vetoshnikova, N-18	
M7v, M7s	V. S. Bespalov, N-18	
M8v, M8s	A. O. Miklashevich, N-18	
M9v, M9s	Lions, NMA	Grey porous travertine
M10v, M10s	E. D. Chaplina, N-18	Pink ovoid granite
M11v, M11s	A. N. Avdulin, N-18	
M12v, M12s	P. V. Skvortsov, N-18	
M13v, M13s	Monument of Unknown, N-18	
M14v, M14s	B. M. Kustodiev, NMA	Wood

Notes: ^1^ NMA—Necropoleis of Master of Art; ^2^ N-18—Necropoleis of the 18th century; ^3^ v—vegetative biomass of the moss; ^4^ s—primary soil under the moss cover.

**Table 2:** Mineral and petrographic characteristics of underlying rocks [41].

No.	Description	Mineral Composition	Assumed Deposit (Geological Age)	Samples of Biodeposits (Table 1)
Marbles				
1	White homogeneous, fine to medium grained marble (statuary marble)	Calcite, quartz	Carrara, Italy (Cretaceous)	M1v, M1s
2	Grey-white banded, heterogeneous, heterogranular carbonate-silicate rock (Ruskeala marble)	Calcite, dolomite, amphiboles (tremolite, hornblende), talc, Fe, Mg—micas, pyroxenes (diopside etc), quartz, apatite	Ruskeala, Karelia, Sortovala region, Russia (Early Proterozoic)	M2v, M2s
Limestones				
3	Grey, porous travertine (Pudostskii stone)	Calcite, dolomite, quartz	Pudostskoe, Leningrad region, Russia (Quaternary)	L1, M9v, M9s
4	Grey-yellow, stratified Limestone (Putilovskaya plita)	Dolomite, calcite, quartz, glauconite	Putilovskoe, Leningrad region, Russia (Ordovician)	M3v, M3s, M3v, M3s, M4v, M4s, M5v, M5s, M6v, M6s, M7v, M7s, M8v, M8s
Granites				
5	Gray, fairly homogeneous fine- and medium-grained rock (Serdobol granite)	Feldspars (microcline, acidic plagioclase), quartz, mica (biotite), pyroxenes and amphiboles	Karelia Sortavala region, Russia (Early proterozoic)	A1, A2
6	Pink coarse-grained porphyritic ovoid granite (Rapakivi granite)		Leningrad region, Karelia, Russia and Finland (Proterozoic)	M10v, M10s, M11v, M11s, M12v, M12s, M13v, M13s

**Table 3:** The average content (wt%) of the main impurity elements in the biodeposits.

Component	Type of Biodeposit			
	I	II	III	IV
Na_2_O	2.0 ^1^	3.0 ^1^	1.7 ^1^	1.5 ^1^
MgO	10.2 ^1^	4.0 ^1^	3.8 ^1^	1.9 ^1^
Al_2_O_3_	10.9 ^1^	16.6 ^1^	11.2 ^1^	8.5 ^1^
SiO_2_	12.3 ^2^	11.1 ^2^	29.4 ^3^	50.6 ^3^
K_2_O	7.6 ^1^	8.0 ^1^	3.9 ^1^	2.1 ^1^
CaO	1.1 ^2^	3.0 ^2^	4.1 ^3^	4.8 ^3^
TiO_2_	0.9 ^1^	1.8 ^1^	0.9 ^1^	0.6 ^1^
MnO	0.1 ^1^	0.2 ^1^	0.1 ^1^	0.1 ^1^
FeO	2.3 ^2^	1.5 ^2^	8.7 ^3^	4.5 ^3^

Notes: ^1^ ICP analysis; ^2^ Electron probe microanalysis; ^3^ X-ray fluorescence analysis.

**Table 4:** The average content of the trace impurity elements (mg/kg) ^1^ in the biodeposits (ICP MS analysis data).

Element	Type of Biodeposits			
	I	II	III	IV
Be	2.0	2.0	1.6	1.4
V	843.0	611.0	219.1	98.4
Cr	801.5	497.0	209.1	77.5
Co	30.3	37.3	25.8	14.9
Ni	100.3	131.8	78.4	38.9
Cu	622.5	924.0	709.6	149.5
Zn	448.0	887.0	676.4	511.6
Ga	18.3	28.5	15.8	11.8
Rb	193.1	191.5	108.9	73.2
Sr	310.5	339.5	342.4	163.0
Y	30.2	39.5	27.1	20.3
Zr	202.0	389.0	56.1	194.2
Nb	14.2	17.3	7.0	8.9
Mo	13.7	16.0	10.5	4.1
Ag	4.5	5.6	2.8	0.1
Sn	41.3	39.3	29.9	10.7
Sb	15.3	14.6	16.9	4.4
Cs	2.8	3.5	2.3	1.6
Ba	1751.0	1385.0	1120.1	631.8
La	57.3	126.6	42.9	28.0
Ce	111.8	238.5	85.1	56.7
Pr	13.2	22.7	9.9	6.5
Nd	48.8	74.2	37.5	26.1
Sm	8.6	11.9	7.1	5.0
Eu	1.5	2.0	1.4	1.0
Gd	7.5	9.9	6.1	4.4
Tb	1.1	1.5	0.9	0.6
Dy	5.7	7.8	5.0	3.6
Ho	1.0	1.5	1.0	0.7
Er	2.7	4.0	2.6	2.0
Tm	0.4	0.6	0.4	0.3
Yb	2.6	3.7	2.4	1.9
Lu	0.5	0.6	0.4	0.3
Hf	4.6	7.9	1.2	4.7
W	27.6	32.9	20.2	10.5
Th	14.6	21.0	11.1	8.3
U	5.6	5.2	7.3	2.6

Note: ^1^ 1 wt% = 10,000 mg/kg.

**Table 5:** Content of the main impurity elements (wt%) in Carrara marble and deposits (dust, moss and primary soil) on its surface.

Component	Marble	Dust	Biodeposits	
			Sam.M1v	Sam.M1s
Na_2_O	0.01 ^2^	3.0 ^1^	1.6 ^1^	1.6 ^1^
MgO	0.17 ^2^	4.0 ^1^	3.7 ^1^	2.0 ^1^
Al_2_O_3_	0.088 ^2^	16.6 ^1^	9.9 ^1^	9.1 ^1^
SiO_2_	0.01 ^2^	11.1 ^2^	36.5 ^3^	50.7 ^3^
K_2_O	0.009 ^2^	8.0 ^1^	3.5 ^1^	2.3 ^1^
CaO	99.0 ^2^	3.0 ^2^	4.3 ^3^	5.2 ^3^
TiO_2_	0.066 ^2^	1.8 ^1^	0.8 ^1^	0.6 ^1^
MnO	0.01 ^2^	0.2 ^1^	0.13 ^1^	0.1 ^1^
FeO	0.019 ^2^	1.5 ^2^	8.2 ^3^	5.2 ^3^

Notes: ^1^ ICP analysis; ^2^ Electron probe microanalysis; ^3^ X-ray fluorescence analysis.
